# Progress in Medicine

**Published:** 1894-11-17

**Authors:** 


### Progress in Medicine.

### INFECTIVE DISEASES.

(*Concluded from page 65.*)

**General Pathology.**—Centanni[1] advances the theory that a common poison, which is the cause of all fevers, is produced by different bacteria, while the varying, clinical symptoms depend on the special conditions of its formation. The activity of bacteria in their pursuit of food leads to their breaking up the substances around them into the elements they require.[2] Thus their staphylococci form abscesses by predigesting the connective tissues through a ferment which has actually been isolated. Others produce a diastatic ferment. The lesions following diphtheria seem, according to S. Martin, to be due neither to the albumose nor the organic acid produced by Loeffler's bacillus, but to a ferment which remains stored up in the spleen, and continues to produce its effects for weeks.

We must distinguish between the albumoses formed in the bacteria themselves from those produced in the surrounding organic fluids. The former are much more stable, and resist even boiling. The albumoses generally cause a fall, rather than a rise, of temperature, and are related to ferments. The toxalbumins derived from snake poison, castor oil, and jequirity seeds, or from the serum of animals, produce focal lesions by the destruction of the cells of the body.[3] Flexner by injecting ricin caused changes in the kidney, spleen, liver, and lymphatic glands of a degenerative type. Thus the liver cells were found to be necrotic, with often fragmentary nuclei. The injection of human or canine serum into rabbits caused changes similar on the whole to those produced by ricin, abrin, or diphtheria toxalbumins, and in both types we may see interstitial proliferation following the acute changes. In the case of tetanus bacilli,[4] Buchner disproved the idea that toxalbumins are formed by a ferment from the tissues of the victim, by growing the bacilli on asparagin without albumen. He was able to produce the usual lesions by injecting the filtered products thus grown. The toxins and antitoxins do not simply neutralise each other if mixed *in vitro* or in the body. The only antagonism is that the antitoxin enables the organism to resist the toxin. Buchner believes that the antitoxin is itself a bacterial[5] product. He shows that the alexin of natural immunity[6] has bactericidal and globulicidal properties, but it is easily decomposed, while the antitoxin produced in artificially immunised animals is more stable, but it has no such properties. Rodel and Courmont precipitated a toxin and an antitoxin from cultures of the staphylococcus, and destroyed the toxin by heating to a degree which had no effect on the antitoxin.

General streptococcus infection[7] is seen under two forms, septicæmia and pyæmia, and is one of the principal causes of death in variola and scarlatina. It arises from the increased virulence of the coccus when grown together with certain saprophytes. Vaughan[8] and Maclintock find that the germicidal constituent of blood serum is a nuclein. This they actually isolated from serum, as it is unaffected by digestion with pepsin. The suprarenal capsules like the liver and other glands are shown by Langlois and Charren to have antitoxin functions,[9] and the phagocytic functions of the lymphoid tissue, for instance, in Peyer's patches are considered of importance by Pekelharing. We may note that Ruffer broached similar views as to the adenoid tissue in the appendix of the rabbit.[10] Madame Schoumoff has conducted further researches on the question of the identity of the streptococcus of erysipelas, scarlatina, and ordinary suppuration by examination of the chemical products of their action, but reliable tests are still to be desired.[11] The great variability of bacteria grown in different media is pointed out by Klein,[12] even in the case of tubercular diphtherial and anthrax bacilli, which under special conditions can be made to revert to or assume forms which show their evident relations to various mycelial fungi. Thus they can be made to produce long branched threads with terminal nodules of protoplasm. Frankland[13] thinks that the evolution of new bacilli is now going on far more rapidly than that of higher organisms, and that fresh pathogenic forms will arise as quickly as we escape from and become immune to the present ones.

**Pyrexia.**—The question of *heat centres* in the human brain in connection with the nervous origin of pyrexia has been reviewed by Dana.[14] He thinks we cannot yet assert that there are any definite foci of the kind, though rises of temperature are more apt to be caused by lesions of the pons than elsewhere. All intra-cranial hæmorrhages are frequently followed by an initial fall and subsequent rise of temperature, especially on the paralysed side. Besides lesions of the pons, those of the cortex are thus characterised, but this is not yet proved of lesions of the basal ganglia in man. He thinks the instances given by Hale White and others do not bear critical analysis, though extensive lacerating lesions, wherever situated, do undoubtedly cause pyrexia. Two cases of pyrexia reaching to 114 deg., followed by the recovery of the patient, are reported in the *Medical Weekly* of May 25th. The value of antipyretics is argued by Kraus to be greater than that of the cold bath, on the ground that their use is shown to be followed by greater heat radiation. He reports[15] that extended examinations of the blood in fever show that the plasma and the size of the red corpuscles does not vary in fever from the normal, while Graham Brown shows that the work actually needed to drive the blood through the vessels in fever is reduced by about one-tenth owing to the decrease in its viscosity.[16] Much discussion has gone on as to the conveyance of the common fevers by sewer gas, and the subject has been reviewed at length by Jacobi, who points out that fewer pathogenic germs have been found in sewer air than in that of houses, and that the moist surfaces of the sewer do not easily give off germs which lie upon them.[17] Sewer air may cause irritation of the throat, which thus becomes a breeding ground for specific germs, but it very rarely conveys such germs itself, the very process of putrefaction destroys them. Professor Notter[18] at Bristol spoke of sewer air as harmless, while sewer gas, which arises from stagnant matter, was dangerous. Much discussion took place as to the best means of ventilating sewers.

**Guaiacol as an Antipyretic.**—A number of reports have been made on this subject. Gilbert[19] used synthetic crystallised guaiacol, which he melted, applied to the skin, and covered with waterproof. He never employed more than twenty-three grains, and found the temperature lowered from 1 to 1·5 deg. C. Cresol produced the same effects. Guinard holds that the action is not produced by absorption through the skin for the internal administration has no such result, but rather by action on peripheral nerves. It only appears in the urine if the vapour is absorbed by the lungs.[20] Later on he found that[21] cocaine, helleborin, spartein, and solanine painted on the skin regulate the temperature. Friedenwald, reviewing the literature of the subject from Sciola onwards, concludes that a fall of from one to four degrees is produced, but this is accompanied by profuse sweating, and sometimes great exhaustion.[22] More than thirty drops should not be applied to commence with, and, in spite of drawbacks, he thinks the plan may be useful where cold baths cannot be employed. Linoissier and Lamois, however, find that it is certainly absorbed by the skin, and that nine times as much appears in the urine when applied externally as when inhaled. It can be found in the urine fifteen minutes after use, and even 55 per cent. can be recovered, so that a patient can be saturated in this manner by the drug without giving it by the mouth.[23] In acute rheumatism and arthritis deformans Desplatz obtained great relief from pain by a dressing of equal parts of guiacol and glycerine,[24] and absorption of pleuritic fluid followed painting with guiacol and iodine. Thayer believes subcutaneous injection produces the same fall of temperature, and finds depressing effects follow every method of using the drug.[25] Dr. Carter, of Birmingham,[26] made 114 trials, and found the temperature fell in 104 cases from 3 deg. to 6 deg. Slight, and occasionally severe, smarting was felt at the place of application, and instances of collapse occurred. After the reduction of temperature a sudden rise is noted by several observers, the explanation of which is not clear, nor is the cause of the fall of temperature itself at all certain. Carter shows that it cannot be the diaphoresis, which may be absent. Kravkoff reports very similar results[27], except that he never observed any collapse in his few cases. C. L. Dana had[28] seldom seen chills or cyanosis from its use in numerous experiments, and confirmed its reported effect in relieving the pain of sciatica. W. Gilman Thompson points out that a fall does not appear to take place if animals are kept in an atmosphere of their normal temperature, otherwise, even in health, their temperature is greatly reduced.[29] Death may occur in the lower animals after painting guiacol on the skin, but he believes the results are obtained by absorption, and not by reflex action. No fall could be detected when all the tissues of the leg were ligated except the sciatic nerve, and conversely when the spinal cord was severed antipyresis occurred quite easily. Hence he discredits Guinard's nerve theory of the action of the drug, and confirms the views of Linossier.

**Rabies.**—According to a paper by Kanthack the prevention and cure of rabies, even after the symptoms have appeared, by the injection of the serum of immunised animals has been the object of Tizzoni and Centanni for some time. It was shown that the serum of rabbits which had been treated by Pasteur's method destroys the virus when mixed *in vitro*, and renders other rabbits immune for six weeks. The antitoxin can be separated with the globulin of the serum, and when obtained from thoroughly immune animals the precipitate will, like the serum, even cut short an attack which has commenced. The next step was to increase its strength by passing it through the bodies of sheep and other large mammals, and at last they got a stock, one part of which will render immune from 25,000 to 50,000 parts of serum. The dose for man will therefore be under 3 cc., or about 3½ grains of the dried powder. If this method proves successful with man it will have many advantages over Pasteur's present plan.

The records of the Pasteur Institute show 1,648 cases treated, with four deaths after a complete course and six before it was ended.[31] The total since the opening now amounts to 14,430 cases, with a mortality of ·5 per cent. Golgi has applied his method to discovering the pathology of the disease,[32] and summarises the changes seen in the nucleus and the cell of the nerve matter and in the inter-vertebral ganglia. These present altogether the picture of a parenchymatous encephalo-myelitis. A severe invasion of hydrophobia occurred at Madeira recently,[33] where it was previously unknown. About 300 dogs died, and the disease was stamped out by shooting and muzzling. Some ten to twenty human beings perished from the disease, as well as many domestic animals. Dr. Goldschmidt deduces from the history of the outbreak the following conclusions, amongst others: First, the severity of the disease in a newly-infected country; and, secondly, the possibility of completely exterminating it by careful muzzling; while certain facts seemed also to show that it is necessary for the disease, when of a mild type, to become intensified by passing through many dogs before it can destroy human beings.

[1] Amer. Jour. Med. Sc., June. [2] B.M.J., Jan. 6th. [3] J. Hopkin's Rep., June. [4] Med. Chron., Vol. XIX. [5] Amer. Jour. Med. Sc., June. [6] B.M.J., July. [7] B.M.J., March 31st. [8] B.M.J., June 9th. [9] Med. Week., May 25th. [10] Med. Week., March 30th. [11] Fortsch. d. Med. 4. [12] New York Med. Jour., July 21st. [13] B.M.J., April 14th. [14] Amer. Jour. Med. Sc., June. [15] Glasgow Med. Jour., July. [16] Int. Med. Mag. [17] New York Med. Jour., July 28th. [18] B.M.J., Aug. 11th. [19] Med. Week., April 20th. [20] Ther. Gaz., March 19th. [21] Lancet, June 30th. [22] New York Med. Jour., April 14th. [23] Practitioner, Sept. [24] Ther. Gaz., May 16th. [25] Ther. Gaz., May 16th. [26] B.M.J., July 7th. [28] B.M.J., July 14th. [29] Med. Rec., June 23rd. [30] Med. Chron., April. [31] B.M.J., April 14th. [32] B.M.J., May 12th. [33] Lancet, May 19th.

